# Mitochondrial Impairment in Healthy Controls With Recent Headache and Multiple Symptoms

**DOI:** 10.7759/cureus.87631

**Published:** 2025-07-09

**Authors:** Beatrice A Golomb, Jessica Y Situ, Gavin Hamilton

**Affiliations:** 1 Department of Medicine, University of California San Diego, La Jolla, USA; 2 Department of Radiology, University of California San Diego, La Jolla, USA

**Keywords:** controls, headaches, mitochondria, symptoms, veterans

## Abstract

Background: Persistent headache and multiple symptoms have each been tied to mitochondrial dysfunction. We assess whether reported recent headache and multiple symptoms (last two weeks) relate to bioenergetics in individuals who do not meet criteria for chronic multisymptom illness (CMI).

Methods: Twenty participants screening negative for CMI rated the presence/absence and severity (0-10) of recent (last two weeks) headache and multiple symptoms and underwent ^31^P-MRS (phosphorus-31 magnetic resonance spectroscopy) to assess the post-exercise phosphocreatine (PCr) recovery time constant (PCr-R). Phosphocreatine is depleted with exercise, and the post-exercise PCr recovery rate depends on the ATP production rate.

Results: Eighteen of 20 participants successfully depressed PCr (on ^31^P-MRS) with exercise, enabling PCr-R assessment. Five participants (28%) reported recent headache. Headache presence was related to PCr-R (seconds): PCr-R mean±SD without headache: 34.6±11.5; PCr-R mean±SD with headache 62.3±22.6, p=0.003. Headache severity (0-10) was correlated with PCr-R (seconds): r=0.79, p=0.0001. Headache presence and severity were related to greater multiplicity of recent nonheadache symptoms: headache severity vs summed nonheadache symptom severity, r=0.68, p=0.001 (correlation to summed symptom ratings without excluding headache, r=0.72, p=0.0004). Longer PCr-R predicted greater summed symptom severity: regression β=0.47 (SE=0.15), p=0.007.

Conclusion: Recent headache and multiple symptoms were related to prolonged post-exercise PCr-recovery in persons screening negative for CMI. Future studies should distinguish among potential foundations for these associations.

## Introduction

Headaches of different kinds, including headache not otherwise specified, cluster headache, and (multiple types of) migraine headache, have shown a relationship to impaired mitochondrial function [1-8]. A relationship of headache to impaired bioenergetics or muscle bioenergetics specifically is additionally supported by the concept of tension headache (sustained muscle contraction, imposing increased muscle energy demand as a cause of headache), relief of headache including migraine by administration of Botox to muscles in the face or head (reducing energy demand for these muscles), and relief of headache, migraine or headache generally, by coenzyme Q10 (increasing cell energy supply) [9,10], including in randomized controlled trials [10] and a meta-analysis [9].

Multiple symptoms are a classic manifestation of impaired mitochondrial function. Thus, for instance, “mitochondrial disorders are caused by a defect in intracellular energy production. In general, these are multi-system disorders … a mitochondrial disease should be considered in the event of dysfunction of more than two organ systems or processes with high-energy requirements” [11]. Nonetheless, since any symptom may only manifest once clinical thresholds for mitochondrial compromise or cell loss are reached (threshold effects [12]), which may take variable time in mitochondrial disorders [13,14], there may be a point at which one or a small number of comparatively mild symptoms are evident.

If persistent multiple symptoms (for at least six months) are absent, gauged by indices designed to capture chronic multisymptom illness, it is unknown whether acknowledgment of recent headache or recent multiple symptoms bears a relation to bioenergetics assessed by the post-exercise phosphocreatine recovery time constant (PCr-R).

Bioenergetic impairment can be assessed noninvasively by the PCr-R. Phosphocreatine is a backup energy source for muscle that is depressed with exercise. Rate of recovery relates to the rate of ATP production [15], and post-exercise PCr-R has been deemed a robust index of mitochondrial energy production in vivo* *[16].

We sought to examine whether acknowledgment of recent headache or recent multiple symptoms bears a relationship to bioenergetics assessed by PCr-R. We incidentally assessed whether sex or ethnicity was related presumptively to bioenergetics assessed by PCr-R in screened “healthy” participants. This may be important for the interpretation of future studies, if the mix of those with versus without headache differs by sex or by ethnicity.

## Materials and methods

Methods

Ethics Statement

The study was approved by UCSD’s HRPP (Human Research Protections Program). All participants gave written informed consent to participate.

Participants

Twenty community-dwelling participants from Southern California were nonveterans recruited as “healthy controls” for a study to assess bioenergetics in a chronic multisymptom health condition (“Gulf War illness”). Age, sex, and ethnicity were selected for consistency with the affected veteran population (predominantly male and Caucasian, mean age 53.4).

Eligibility required that participants have not met the symptom eligibility portion of the criteria for Gulf War illness (GWI)/“chronic multisymptom illness”: specifically, they could not meet either the CDC or Kansas [17] Gulf War illness symptom criteria [18]. Symptom criteria are as follows: CDC criteria require the presence of one or more symptoms in each of at least two of the three domains of fatigue, musculoskeletal, and mood-cognitive [18]. Kansas GWI criteria require that veterans have multiple symptoms within a qualifying domain, and/or symptom(s) of at least moderate severity, in at least three of the six domains of neurological-cognitive-mood, fatigue/sleep problems, respiratory, pain, gastrointestinal, and skin [17] using a structured assessment tool. To qualify for either set of criteria, symptoms must have persisted over at least the last six months.

Medical and Medication Exclusion Criteria

Participants could not have Kansas exclusion criteria, i.e., other conditions, like multiple sclerosis, lupus, diabetes, and heart disease, that could contribute to symptoms that might lead to symptom qualification [17]. Persons currently on agents known to produce mitochondrial toxicity (such as fluoroquinolones [19-21] or amiodarone [22]) were excluded. Participation requires eligibility to undergo ^31^P-MRS (e.g., no interfering metal, weight <300lbs) and the ability to follow simple directions. Those with a history of welding or other occupations in which metal fragments can enter the eye underwent an orbital x-ray to exclude metal in the eye prior to ^31^P-MRS participation.

Assessments

Recent Symptoms

Participants, on the date of ^31^P-MRS, completed a questionnaire inquiring about symptoms in the last two weeks (hereafter often referred to as “recent symptoms”). This included headache, as well as 123 other symptoms (Table 7). Recent symptoms were self-rated as present-absent, and severity was self-rated from 0 to 10 (where 10 is the worst possible severity), with a rating of 0 conferred for those who designated the symptom as absent. (Again, the presence of multiple symptoms that persisted for at least six months had been queried as part of a screening interview as above, and presence of such symptoms adequate to qualify for CMI/GWI led to exclusion.)

PCr-R Assessment

Spectra were acquired on a 3 Tesla GE Signa EXCITE HD scanner (GE Healthcare, Waukesha, WI). The 1H signal was acquired using the body coil for the collection of multiplanar localization images and shimming. Subjects were scanned in the supine position. The ^31^P-MR spectra were collected with a 5” surface coil, using a slice-selective free induction decay sequence with a repetition time of 3 sec. The spectra sampling interval was 0.2 msec, with 2048 data points collected. Spectra were collected at rest with 128 signal averages. The coil was placed under the calf. A spectrum was collected every repetition time during two minutes of rest (resting muscle spectra), then through five minutes of exercise (repetitively pushing a pedal with the foot, similar to depressing a car pedal) and six minutes of recovery. Study staff “cheered” the participant to continue foot pedal depression during the exercise time to increase the prospects of achieving adequate exercise of the desired musculature and PCr depression. An exponential was fitted to the post-exercise phosphocreatine recovery curve, and the time constant on the exponential was the designated PCr-R (assessed in seconds). Failure to achieve successful PCr depression precluded fitting of an exponential and thus determination of PCr-R.

Analyses

Descriptive statistics characterized participants. The relation of headache presence and headache severity (last two weeks) to PCr-R was assessed by T-tests (for headache presence), correlation, and regression with robust standard errors. The same analysis approaches assessed whether (recent) headache presence and severity were related to recent nonheadache symptom number and summed (nonheadache) symptom severity. PCr-R relation to symptom number and summed symptom severity was assessed. Scatter plots with a fitted regression line were used to depict the relation of symptom number and summed symptom severity to PCr-R (with and without exclusion of persons citing no symptoms).

To more broadly assess potential sources of variance in PCr-R, we also examined (exploratory) whether sex or ethnicity appeared to relate to PCr-R in this sample, with white male participants as the comparator group, excluding participants who cited headaches (to more clearly examine the possible impact of sex and ethnicity).

Multiple assessment adjustment rests on the presumption that chance is the first-order explanation for a significant finding [23]. Since there is prior evidence supporting a relation of impaired bioenergetics (here, prolonged PCr-R) to headache and multiple symptoms, if significance is seen in these relationships, chance is not the first-order explanation; therefore, multiple assessment adjustment was not indicated (or undertaken). In contrast, for the exploratory analysis by sex and ethnicity, observed relationships merit follow-up in future samples; findings in these domains are clearly characterized as highly provisional.

## Results

PCr depression was successfully achieved in 18 of the 20 participants, permitting PCr-R to be determined by fitting an exponential to the recovery data.

**Figure 1:**
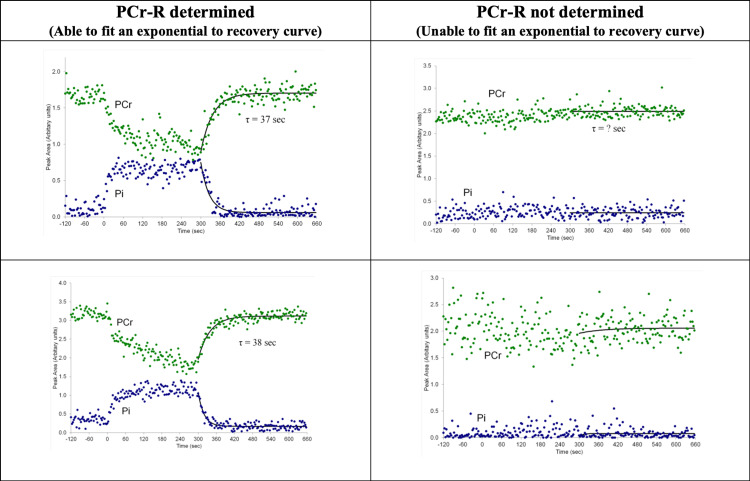
Successful (Left Column) and Unsuccessful (Right Column) Exercise Scans. PCr-R: Phosphocreatine recovery time constant

Examples of successful and unsuccessful exercise scans are shown in Figure 1. No significant difference was found between the pre-exercise (resting) and post-exercise pH values. This was true in the total sample and in cases and controls assessed separately. Since pH did not significantly differ in the post-exercise relative to the pre-exercise (rest) phase, this enables PCr-R to serve as a proxy for ATP production.

Tables 1, 2show characteristics of these participants, including all and those contributing PCr-R to subsequent assessments. Five participants reported recent headaches, all of whom had successful PCr-R determination.

**Table 1:** Participant Characteristics – Binary Variables. PCr-R: Phosphocreatine recovery time constant

	All ( N = 20)	With PCr-R ( N=18)
	Percent	Percent
Male	90.0	88.9
Caucasian	85.0	83.3
Headache present	25.0	27.8

**Table 2:** Participant Characteristics – Ordinal and Continuous Variables. *124 symptoms were queried. There were 38 symptoms for which at least one of the “healthy controls” that are the focus of this study, cited presence of the symptom recently (last two weeks). †Numbers are N/A for PCr-R for the “All” column, because only those with PCr-R can contribute values for PCr-R. PCr-R: Phosphocreatine recovery time constant

	All ( N = 20)	With PCr-R ( N=18)
	Mean ± SD (Range)	Mean ± SD (Range)
Age	54.1 ± 6.67 (43-66)	54.5 ± 6.82 (43-66)
Headache severity (0-10)	0.72 ± 1.33 (0-4)	0.80 ± 1.38 (0-4)
# Symptoms including headache*	2.90 ± 3.71 (0-11)	3.17 ± 3.82 (0-11)
Summed symptom rating, including headache	11.7 ± 16.2 (0-49.8)	12.9 ± 16.6 (0-49.8)
# Symptoms excluding headache*	2.65 ± 3.39 (0-10)	2.89 ± 3.50 (0-10)
Summed symptom severity, excluding headache	11.0 ± 15.2 (0-46.6)	12.1 ± 15.7 (0-46.6)
PCr-R†	N/A	42.3 ± 19.4 (11.5-90.9)

The PCr-R was markedly higher on average in those with headache (Table 3).

**Table 3:** Headache Presence (vs Absence) in the Last Two Weeks Predicts PCr-R. *Regression with robust standard errors. †T-test with unequal variances is also significant. PCr-R: Phosphocreatine recovery time constant

	Regression: Prediction of PCr-R by headache presence*		T-test of difference in PCr-R by headache presence†		
	β (SE)	P	No Headache, PCr-R, Mean (SD)	Headache, PCr-R, Mean (SD)	P
Controls	27.6 (10.1)	0.015	34.6 (11.5)	62.3 (22.6)	0.003

Table 4 shows that greater headache severity ratings also significantly predicted more prolonged PCr-R.

**Table 4:** Headache Severity Rating (0-10 rating) Predicted PCr-R (seconds). *Regression with robust standard errors. Headache severity scored 0-10. PCr-R in seconds. P-values <0.05 are considered statistically significant and are shown in bold. PCr-R: Phosphocreatine recovery time constant

	Regression: Prediction of PCr-R by headache severity*		Correlation of PCr-R with headache severity	
	β (SE)	P	r	P
Controls	11.0 (2.05)	<0.001	0.79	0.0001

The three highest PCr-R values (the most delayed PCr-recovery) were each in participants who reported the presence of recent headache (and comprised three of the five who did report headache). Moreover, these were the three controls, out of the five who reported recent headaches, who scored their headache severity the highest. PCr-R values for these three more-severe-headache participants were markedly higher than typical PCr-R values of persons without headache (mean <35 seconds), as shown in Table 5.

**Table 5:** Highest PCr-R Values Were in Participants with Highest Headache Ratings. PCr-R: Phosphocreatine recovery time constant

Headache Rating (0-10)	PCr-R (seconds)
4	90.9
3.2	73.6
3.1	66.6

Table 6 shows that greater (two week) headache severity ratings were also tied to a greater number of and summed severity rating of (two week) nonheadache symptoms.

**Table 6:** Headache vs Summed Number and Summed Severity of Assessed Nonheadache Symptoms. *These analyses use a number of symptoms excluding headache, and summed symptom ratings excluding headache rating. Headache presence correlations, each 0.69 (p=0.0008), differ in the third digit after the decimal point. †Unequal variance t-test also significant assessing headache presence against each of the nonheadache symptom indices. Use of the unequal variance test is not indicated for symptom number but is appropriate for summed ratings (p = 0.019). P-values <0.05 are considered statistically significant and are shown in bold.

	Headache Severity				Headache Presence						
	Correlation		Regression		Correlation		Regression		T-test†		
									No (Headache)	Yes (Headache)	
Variable*	r	P	β (SE)	P	r	P	β (SE)	P	Mean (SD)	Mean (SD)	P
Number symptoms	0.66	0.002	1.67 (0.48)	0.003	0.69	0.0008	5.26 (1.37)	0.001	1.33 (2.41)	6.6 (2.88)	0.0008
Summed symptom ratings	0.68	0.001	7.70 (2.36)	0.004	0.69	0.0008	23.6 (6.75)	0.003	5.12 (2.63)	28.7 (14.7)	0.0008

The median number of nonheadache symptoms was also significantly greater for those citing recent headache, and all five of those citing headache had nonheadache symptoms exceeding the median (p=0.008 with continuity correction, p=0.002 without). Similarly, the median summed nonheadache symptom rating was significantly greater in those citing recent headache, and those with headache had summed nonheadache symptom ratings exceeding the median in every case (chi-2 p=0.039 with continuity correction, p=0.010 without).

The two participants with the highest recent headache ratings also had the highest summed symptom ratings (last two weeks), 49.8 and 37.7, to which headache contributed 3.2 and 4 points, respectively.

Longer PCr-R predicted a greater number and summed intensity of recent symptoms (Table 7). These relationships were less strong than the relationships of PCr-R to headache.

**Table 7:** Greater Number of Symptoms, and Greater Summed Symptom Ratings, Significantly Related to Longer PCr-R. *Robust standard errors. P-values <0.05 are considered statistically significant and are shown in bold. PCr-R: Phosphocreatine recovery time constant

	Correlation to PCr-R (seconds)		Regression predicting symptoms by PCr-R*	
Variable	r	P	β (SE)	P
Number symptoms	0.50	0.034	0.099 (0.032)	0.008
Summed symptom ratings	0.54	0.020	0.47 (0.15)	0.007

The highest summed symptom ratings (49.8 and 37.7) were in those with the most delayed phosphocreatine recovery (PCr-R of 73.6 and 90.9 seconds, respectively).

Of note, the participant with the highest PCr-R (the highest headache score, the second highest summed symptoms), a Caucasian male, was also the sole participant to check the presence of chemical sensitivity (assessed on the 31P-MRS visit day).

**Figure 2:**
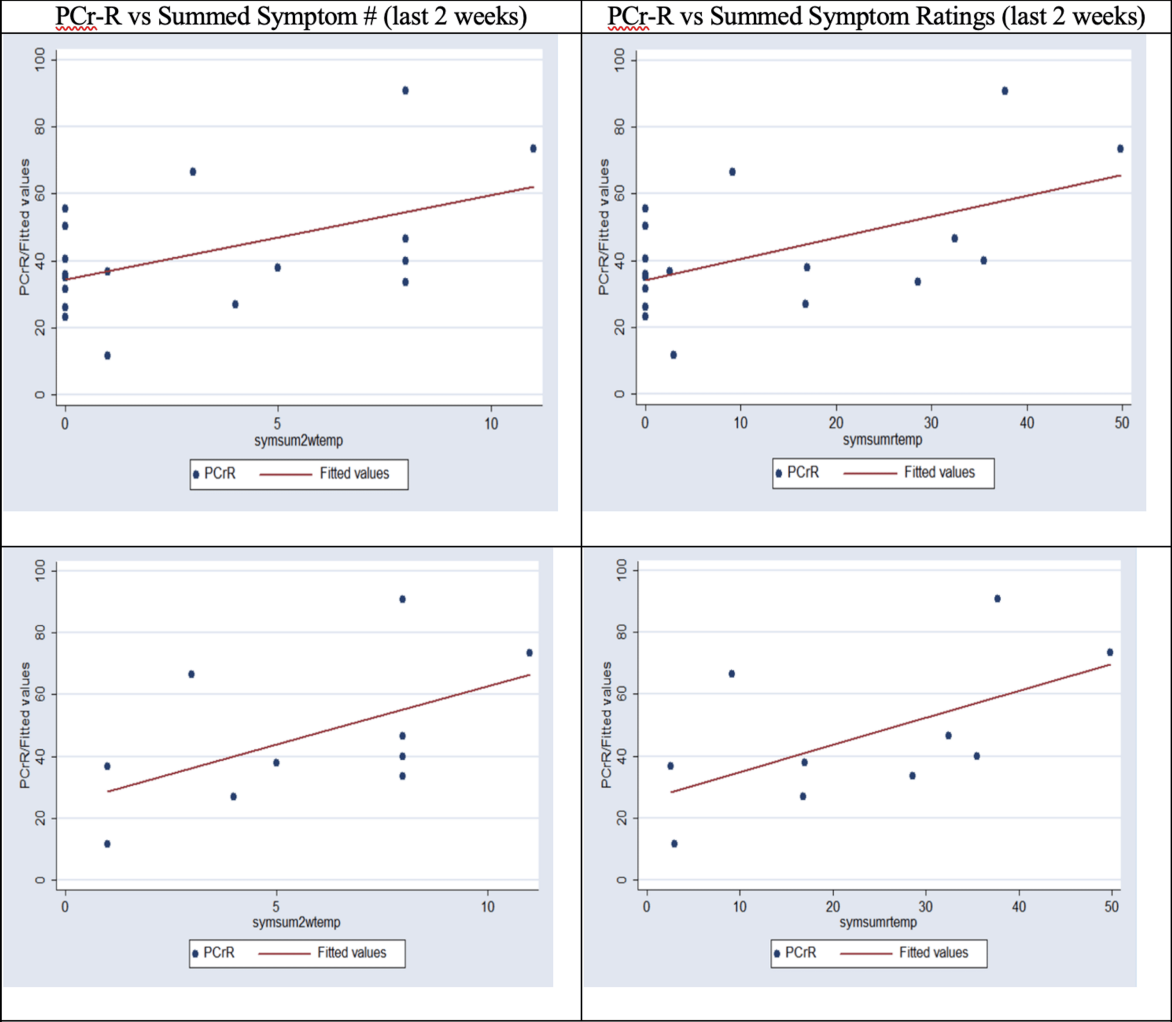
PCr-R vs Summed Symptoms (Last Two Weeks) PCr-R as a function of summed number of symptoms (in the last two weeks), on the left; and summed symptom ratings (for symptoms present in the last two weeks), on the right. Top graphs include all. Bottom graphs exclude those with “0” symptoms acknowledged. PCr-R: Phosphocreatine recovery time constant

Figure 2 shows PCr-R as a function of summed symptom number and summed symptom ratings (last two weeks). The upper row shows the scatterplot and regression line, including those with summed symptom scores of zero. The lower row excludes those with summed symptom scores of zero.

As noted in the methods, we also examined whether sex or ethnicity appeared to relate to PCr-R, excluding participants who cited headaches, to better understand the possible impact of sex and ethnicity on results. Values in white male participants were very closely similar to those of male mostly-white healthy controls in a previous study [24], 30.3 seconds (SD 9.20) for this study; 29.0 seconds (SD 8.7) for the prior study with an independent sample. In this sample, excluding those who cite recent headache from all groups, those who were female, or nonwhite had apparently slower PCr recovery after exercise (~50% longer PCr-R): female Caucasian participants 45.5 seconds (SD 7.06) and non-Caucasian male participants 43.5 seconds (SD 17.1), but based on only two per group for Caucasian female participants and nonwhite male participants. Assessing white male participants’ PCr-R against PCr-R of combined others, i.e., Caucasian female participants plus nonwhite male participants (PCr-R mean 44.4 seconds, SD 10.7), the difference was significant (2-sided p=0.031). This non-prespecified analysis is based on quite small numbers and must be viewed as emphatically provisional, but it merits to note that every white male participant had lower values of PCr-R than either female participant, or than one of two nonwhite male participants, considering in these analyses only participants without headache.

## Discussion

Recap of findings

Five of 18 “healthy controls” who did not qualify as having chronic multisymptom illness by two commonly used screening criteria, cited the presence of recent headache (last two weeks), and rated the severity of recent headache. Both the presence and severity of recent headache were each tied to a markedly (and statistically significantly) longer phosphocreatine recovery time constant after exercise (PCr-R), signifying presumed bioenergetic impairment [16] (mitochondrial impairment by a broad definition). The three participants citing headache as most severe had the three highest PCr-R values among the 18 in whom PCr-R was determined. Recent headache and higher PCr-R were each also significantly tied to recent symptom multiplicity (not due just to headache).

Of note, values for healthy male participants without headache were closely similar to those of the mostly white all male healthy controls (without headache) in a prior study [24]. As a highly provisional finding, based on small numbers (and not hypothesis driven), the PCr-R values in healthy female participants and non-Caucasian male participants in this sample were materially higher than in healthy white male participants. Many factors could account for this (including the play of chance, different factors that may lead women and minorities to participate in studies, or typically higher or more varied PCr-R values in women and minorities, among many other possibilities). The finding was not grounded in a hypothesis and is based on small numbers, so it is highly provisional and hypothesis-generating. It may support the well-known benefits of sample homogeneity in limiting variance and enhancing study power. But it also supports the need to study women and minorities precisely because they may be different. Without expanding sample characteristics beyond the earlier study’s homogeneous characteristics, the potential to identify differences and to caution about features that may be sources of heterogeneity is obviated.

Fit with literature

Headache has been tied to impaired mitochondrial status/bioenergetics in a number of studies [1-8]. One example, among numerous mitochondrial and bioenergetic findings in association with headache and/or migraine, found a relation of migraine to nuclear encoded mitochondrial proteins (most mitochondrial proteins are encoded by nuclear DNA), which in a replication sample were found to implicate genes governing phosphorylation, fatty acid metabolism, and oxidative demethylation [25]. Headache may commonly involve muscle energy supply-demand imbalance (not necessarily exclusively of mitochondrial origins). Evidence in support of this comes from the nature of tension headache (persistent muscle contraction imposes increased energy demand), alleviation of headache with Botox applied to facial muscles (reducing energy demand - expressly, indeed, in muscle in the head), and alleviation of headache with mitochondrial supportive and antioxidant supplement coenzyme Q10 in a meta-analysis of randomized controlled trials for migraine [9]. Of relevance to the strong connection of bioenergetics/mitochondrial compromise to headache, and the more moderate connection to multiple symptoms, coenzyme Q10 was reported to significantly alleviate multiple symptoms, but especially headache in a randomized control trial of multisymptom GWI [10]. (Multiple symptoms in that case were persistent.) Regarding PCr-R specifically, prolonged PCr-R in muscle has been previously reported in association with migraine with aura (in adults [26] and children [27]) and cluster headaches [28] but has not been previously reported in association with headache in a generally recruiting (nominally healthy) sample unselected for any specific headache class. It is noteworthy that other forms of pain beyond headache have also been tied to mitochondrial impairment, including myalgia in the absence of creatine kinase elevations that can arise with statin therapy [29], which can lead to dose-dependent reductions in coenzyme Q10 [30] (a mitochondrial electron transport chain coenzyme). Additionally, previous studies have not examined mitochondrial impairment in relation to multiple concurrent symptoms, in a sample not selected for health problems (and indeed in principle, selected for absence of chronic multisymptom illness, or at least absence of illness that would qualify by Kansas or CDC symptom criteria).

It has been asserted that the presence of multiple symptoms, particularly in the absence of a known diagnosis expected to cause such, should lead to mitochondrial impairment being considered [11]. In this sample, recent headache and recent multiple symptoms were each tied to bioenergetic impairment, with the relation of multiple symptoms to bioenergetic impairment not explained by the contribution of headache to the multiple symptoms. The relation of recent multiple symptoms to prolonged PCr-R comports with the often-multisymptom/multisystem nature of bioenergetic/mitochondrial impairment [11,31]. Apropos of this, it has been reported that patients with chronic fatigue illness, using a multisymptom criterion similar to that used for GWI, were tied to mitochondrial impairment, using an innovative ATP profile test that gauged stages of ATP production in neutrophils [32]. Gulf war veterans, with chronic multisymptom illness, have shown bioenergetic impairment on PCr-R [24,33], and with mitochondrial assessment on biopsy [34] (and severity of which has been tied to mitochondrial haplogroup U [35]). This study’s tie of multiple symptoms to greater PCr-R also echoes a call for recognition that depression with “somatization” symptoms generally implicates mitochondrial impairment [36,37]. Additionally, mitochondrial impairment has been shown in association with a range of “functional” somatic disorders, encompassing the likes of complex regional pain syndrome and non-specific abdominal pain [38].

This study’s finding that the participant with the highest PCr-R (who had the highest headache score, and second highest summed symptoms) was also the sole “healthy control” to indicate the presence of chemical sensitivity is of possible interest. Chemical sensitivity is among multisymptom “overlap conditions” that are each present at higher rates when other multisymptom conditions are present, such as chronic fatigue [17,39-43], fibromyalgia [17,41,42,44], and/or Gulf War illness [33,34,43], conditions each of which have been tied to impaired cell energy. We have previously conjectured that shared mechanisms among these conditions include mitochondrial impairment [45]. Indeed, drug/chemical intolerance is a common problem in mitochondrial conditions, where it is noted that avoiding certain drugs is often more beneficial than applying apparently indicated drugs [46]. Our work in Gulf War veterans directly connects (shows a strong relationship between) the themes of chemical sensitivity and propensity to experience drug/chemical adverse effects [47]. Further support for a tie between mitochondrial impairment and chemical sensitivity comes from evidence supporting a relation between chemical sensitivity and a gene variant of the chief mitochondrial antioxidant, manganese superoxide dismutase (also called SOD2) [48] and our preliminary data in Gulf War veterans support this association [49]. Additionally, it was recently shown that summed symptom severity in veterans with GWI (with GWI in turn tied to chronic fatigue syndrome [17,39-43], fibromyalgia [17,41,42,44], irritable bowel syndrome [40,44] and multiple chemical sensitivity [45]) related to severity of mitochondrial impairment assessed from muscle biopsy [34] - as well as to mitochondrial haplogroup U [35], which was previously tied to conditions that implicate mitochondrial impairment and oxidative stress [50].

These findings have critical implications for somatic symptoms that, particularly when they encompass diverse classes, have often been historically ascribed to psychogenic/somatoform causes, likely in part because tests for mitochondrial impairment have not been readily available (and most physicians are not trained to consider mitochondrial foundations for symptoms).

Mechanisms

These participants were chosen for not having multiple chronic symptoms. There is nothing inherently contradictory about the presence of recent symptoms in the absence of chronic or persistent symptoms. But the recent symptoms tied to bioenergetic impairment do raise important questions. Were symptoms and bioenergetic impairment both transitory? In that case, the same subjects at a future time may lack recent symptoms and exhibit PCr-R values that are more typical. Might a recent exposure or event have triggered chronic changes in bioenergetics and symptoms? In that case, participants at a later date would report persistent or recurrent symptoms and continue to show elevated PCr-R. Might recent headache reflect recurrent but intermittent headache, not characterized by participants as persistent, in the presence of bioenergetic deficit? If a problem may hinge on the interpretation of the word “persistent” in the query about chronic symptoms, screening tools meant to identify cases may require rewording to secure controls without persistent problems manifesting as intermittent multiple symptoms. Yet another possibility is that participants may be aware, from recruitment materials, that they are screening to be healthy controls in a study. Those who are interested in participating may, consciously or unconsciously, shade their interpretation of their own symptoms in their responses to screening inquiries, to influence eligibility. Future studies can assess several such possibilities, such as by reassessing both headache and PCr-R in persons who acknowledged recent headache (but denied chronic headache) at several later time points, including, if possible, at times when recent headache is cited as absent.

A further question is raised from the (highly provisional) finding of apparently higher PCr-R values in women and minority participants without headache than in white men without headache. Because these findings are based on small groups and were not prespecified, findings should be viewed with caution and construed as hypothesis-generating only. Future studies should seek to assess whether comparatively longer PCr-R is present in these groups. This could reflect the play of chance, differences in the reason for study participation as controls in women and minorities relative to white men, or other factors. Among other speculative factors, women are more vulnerable to many toxins and exposures, including common mitochondrially toxic agents [51-56]. Differences in body size [56] or differences in detoxification mechanisms/ slower clearance may contribute [55,57]. Minorities may be more often exposed to toxins (the basis of environmental justice campaigns). Factors such as these could contribute to higher PCr-R values in those groups (if subsequent studies suggest these are indeed higher), and there may be a delay to phenotypic expression where mitochondrial problems have been introduced (e.g., delay to cell loss). Symptoms in many mitochondrial conditions, including heritable ones, can be of late onset [13,14] and may be delayed until so-called (phenotypic) “threshold effects” are reached [12].

## Conclusions

The number of participants in this analysis was modest, and no minimum number with a recent headache was required. However, the large effect size (offsetting the small sample size to achieve quite strong significance) coupled with fit of findings with existing evidence tying headache to mitochondrial impairment adds to authority of the findings. As above, it is unclear whether reported symptoms in the absence of chronic symptoms reflect transitory or new-but-sustained features. If symptoms are transitory, it is also uncertain whether bioenergetic impairments resolve alongside symptom resolution. Future studies will be needed to clarify this.

This study supports a relationship between headaches as well as multiple symptoms and impaired bioenergetics, extending this to the setting in which symptoms are reportedly recent but not persistent. These findings cohere with and extend other evidence tying headache and multiple symptoms to bioenergetic compromise, including reported benefits to headache of treatments that enhance cell energy.

## Appendices

**Table 8:** 124 Symptoms Present in the Past Two Weeks

Symptoms			
1.	Aches/pains	63.	Fits or convulsions
2.	Joint Pain	64.	Flare up of acne
3.	Muscle pain	65.	Flatulence (excessive farting)
4.	Headache	66.	Gas, indigestion, or other stomach problems
5.	Tiredness	67.	Hair Loss
6.	Sleep Problems	68.	Hand shaking
7.	Having too little energy to start doing things	69.	Head colds
8.	Muscle Weakness or Fatigability	70.	Hearing Loss
9.	Post-exertion fatigue	71.	Heartburn
10.	Irritability	72.	Hopelessness
11.	Impatience	73.	Immediate memory problems (eg…)
12.	Anxiety	74.	Increased Infections, other
13.	Having to go back and check that you have done things	75.	Increased Upper Respiratory Infections
14.	Trouble recalling words or names when you want	76.	Instability or imbalance while walking
15.	Attention or Concentration Problems	77.	Intolerant to small amounts of alcohol
16.	Difficulty remembering what you were doing or where you were going	78.	Itching Skin
17.	Difficulty grasping the meaning of what you read	79.	Joylessness
18.	Cold hands or feet	80.	Leg Cramps
19.	Dry Skin	81.	Losing Sleep due to worry
20.	Ringing in ears	82.	Loss of Confidence
21.	Abdominal Bloating	83.	Loss of Coordination
22.	Abdominal Pain or Cramping	84.	Loss of enthusiasm for and enjoyment of life
23.	Abnormal Bleeding	85.	Loss of interest in sex
24.	Altered Temperature Regulation	86.	Loss of interest in socializing
25.	Blackouts	87.	Memory flashbacks
26.	Bleeding Gums	88.	Memory problems
27.	Blood Glucose Decrease	89.	Mood changes
28.	Blood Glucose Increase	90.	Mouth ulcers
29.	Blood Pressure Decrease	91.	Muscle twitching
30.	Blood Pressure Increase	92.	Nausea
31.	Boils or Abscess	93.	Needing to urinate at night, more than once
32.	Breast Enlargement (men)	94.	Night sweats
33.	Changes in Sexual Function	95.	Nightmares
34.	Chemical Sensitivity	96.	Nosebleeds
35.	Chest Infections	97.	Palpitations or feeling of irregular heartbeat
36.	Clumsiness	98.	Peripheral Neuropathy (e.g., numbness, tingling, burning in extremities or elsewhere)
37.	Constipation	99.	Persistent cough when do not have a cold
38.	Coordination or Balance Problems	100.	Phlegm or sputum
39.	Depressed Mood	101.	Poor appetite
40.	Diarrhea	102.	Problems buttoning your clothes
41.	Difficulty Achieving Orgasm	103.	Rash or sores or Skin problems
42.	Difficulty Getting Aroused/ getting an erection	104.	Short Temper
43.	Difficulty staying aroused/ keeping an erection	105.	Sinus Congestion
44.	Difficulty lifting down an object from just above your head	106.	Sleep change
45.	Difficulty standing from a chair	107.	Shortness of Breath
46.	Difficulty grasping the meaning of what you read	108.	Sleeping or napping during the day
47.	Difficulty with performing a job or usual functions	109.	Slurring your words
48.	Dizziness	110.	Sore or swollen glands in neck
49.	Double vision	111.	Sore throat
50.	Dry Eyes	112.	Sudden changes of mood
51.	Dry Mouth	113.	Swelling of Extremities
52.	Ear Infections	114.	Thinking you are a worthless person
53.	Excessive hunger or food cravings	115.	Toothache
54.	Excessive Sweating	116.	Tremors
55.	Falls	117.	Vaginal Dryness/Atrophic Vaginitis (women)
56.	Feeling detached from others	118.	Vertigo (or feeling of vertigo)
57.	Feeling of heaviness in your chest	119.	Veteran or partner feels a burning sensation after sex
58.	Feeling sick	120.	Vision changes
59.	Feeling spacey or disconnected	121.	Weight Decrease
60.	Feeling stiff	122.	Weight Increase
61.	Feeling unrested after sleep	123.	Other physical symptoms
62.	Fevers	124.	Other symptoms
